# Severe ischemic gastritis caused by chronic mesenteric ischemia

**DOI:** 10.1590/1677-5449.202300022

**Published:** 2023-08-11

**Authors:** Bruno Moraes Ribas, Eduardo Camargo Rebolho, Guilherme Figueiró Ferronatto, Pedro Henrique Bragato, Hugo Genki Kagawa Akahane, Eduardo José Brommelstroet Ramos, Thienis Maria da Costa Lima, Barbara D’agnoluzzo Moreira

**Affiliations:** 1 Hospital Nossa Senhora das Graças - HNSG, Curitiba, PR, Brasil.; 2 Hospital Marcelino Champagnat, Curitiba, PR, Brasil.; 3 Hospital São Vicente - HSV, Curitiba, PR, Brasil.; 4 Universidade Federal do Paraná - UFPR, Curitiba, PR, Brasil.; 5 Hospital de Clínicas de São Paulo - HC-SP, São Paulo, SP, Brasil.

**Keywords:** mesenteric ischemia, mesenteric vascular occlusion, atherosclerosis, endovascular procedures, gastritis

## Abstract

Ischemic gastritis is a rare illness caused by localized or systemic vascular insufficiency. This condition is rarely seen in medical practice due to the vast arterial collateral blood supply to the stomach through the celiac trunk and superior mesenteric artery and also because other etiologies are much more frequent. The classic presentation of chronic ischemia is comprises the triad of postprandial pain, weight loss, and abdominal bruit. Intervention is indicated in symptomatic patients and endovascular treatment is an alternative to surgery in patients with high comorbidity that offers good results. We report a case of a 71-year-old female patient with severe ischemic gastritis with ulcers and bleeding caused by chronic mesenteric ischemia with occlusion of the celiac trunk and inferior mesenteric artery and critical stenosis of the superior mesenteric artery. The diagnosis was confirmed by imaging, and the patient underwent endovascular treatment. This is a rare condition that is difficult to diagnose and treat and a multidisciplinary team is needed for proper management.

## INTRODUCTION

Mesenteric ischemia can be secondary to arterial or venous insufficiency, or may be non-occlusive.^1^ Predisposing factors include cardiovascular disease, smoking, arterial fibrillation, hypercoagulability, and vasculitis.^2^

The acute form is defined as an abrupt interruption of blood supply and emboli of the superior mesenteric artery (SMA) is the most common cause (50%).^2^ The main symptom is intense abdominal pains, disproportional to physical examination findings, with onset within minutes in embolic cases and hours in atherothrombotic presentations.^2^ As the condition progresses, the systemic manifestations become predominant, with tachycardia, hypotension, fever, and leukocytosis.^2^

The chronic form occurs when at least two of the major arteries are compromised.^2^ The condition primarily affects females, aged 40 to 70 years, and there is evidence of atherosclerotic disease in 2/3 of cases.^2^ It often leads to weight loss since patients become scared to eat because eating can provoke nausea, vomiting, mucosal bleeding, and anemia.^2^

Ischemic gastritis is a rare presentation and its etiology can be local or systemic vascular insufficiency. Atherosclerosis with celiac trunk involvement is the most common cause (75%).^2,3^ Among systemic variants, 25% of cases are caused by hypoperfusion, with a particular emphasis on septic shock.^2,3^ Surgical treatment is indicated in symptomatic cases, which can alter the natural course and improve prognosis.^1^

The research protocol was approved by the Ethics Committee at our institution (CAAE:60299722.8.0000.0096, decision number 5.697.538).

## CASE DESCRIPTION

The patient was a 71-year-old woman, weighing 37 kg, and complaining of colic-like abdominal pains, chronic diarrhea, and approximately 10 kg weight loss over the course of 1 year. Pain occurred 2-3 times a day, was most intense in the right hypochondrium, and was directly related to eating. The patient was hypertensive and an ex-smoker, having quit smoking 20 years previously. She had a history of open cholecystectomy and appendectomy. She was thin, with a concave, flaccid, and painless abdomen, with no palpable masses, and with a systolic murmur in the epigastrium.

**Figure 1:**
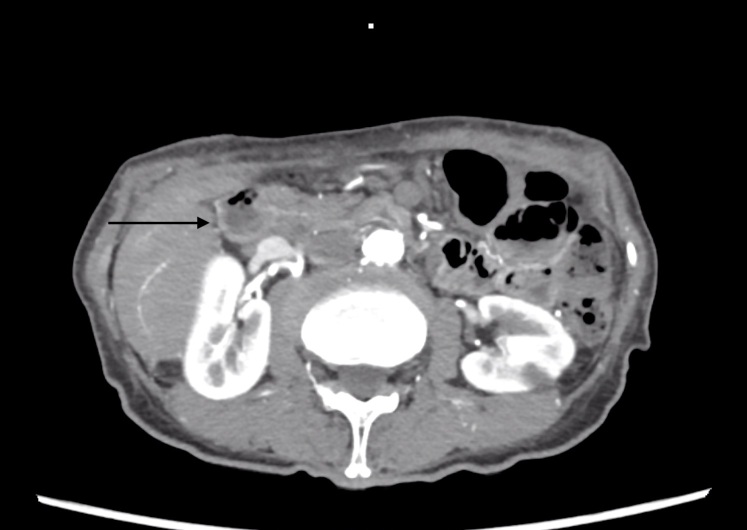
Abdominal tomography showing the 5mm hypotension nodule in the duodenal ampulla.

**Figure 2:**
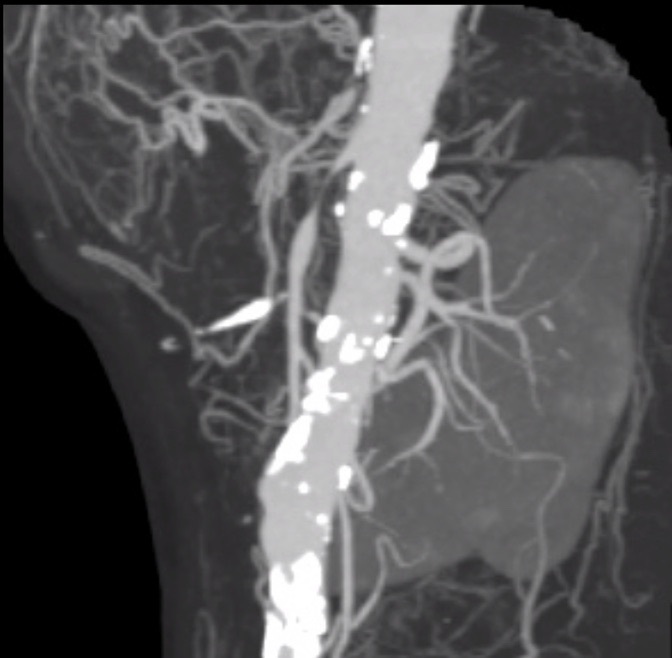
Angiotomography of the abdominal aorta and branches, showing occlusion of the celiac trunk with distal filling via collaterals, and critical stenosis of the superior mesenteric artery and inferior mesenteric artery.

**Figure 3:**
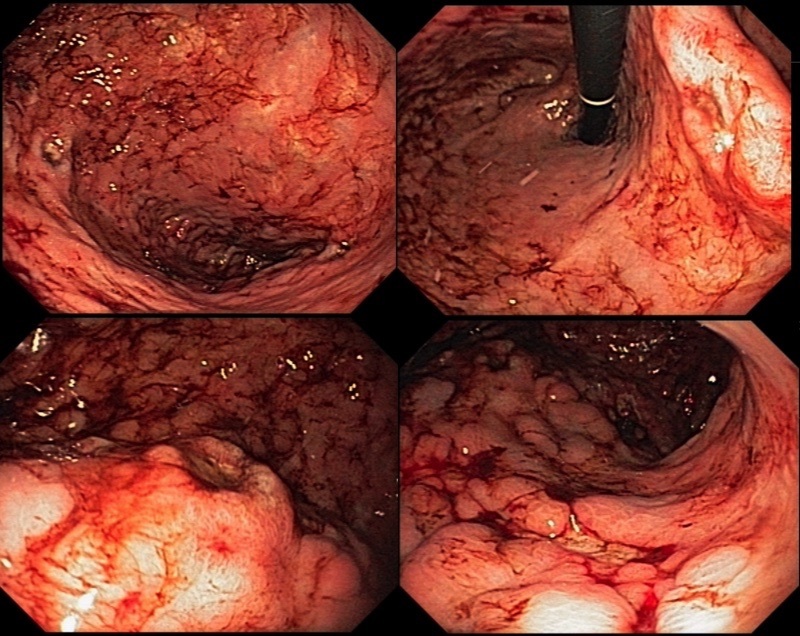
Upper digestive endoscopy showing multiple, bleeding, superficial ulcers with irregular margins and areas of pallor.

Abdominal tomography with contrast showed a hypodense nodule in the duodenal ampulla measuring 5 mm (Figure 1). There was occlusion of the celiac trunk (CTr), but with distal refilling via collaterals, occlusion of the inferior mesenteric artery and critical SMA stenosis, which had a 1mm lumen (Figure 2). A hypothesis of carcinoma of the duodenal ampulla or biliary ducts was ventured and she underwent an upper digestive endoscopy (UDE), demonstrating multiple superficial gastric ulcers with irregular margins and bleeding along the body of the stomach, with areas of pallor, suggestive of ischemic gastritis, and normal duodenal papilla (Figure 3).

**Figure 4:**
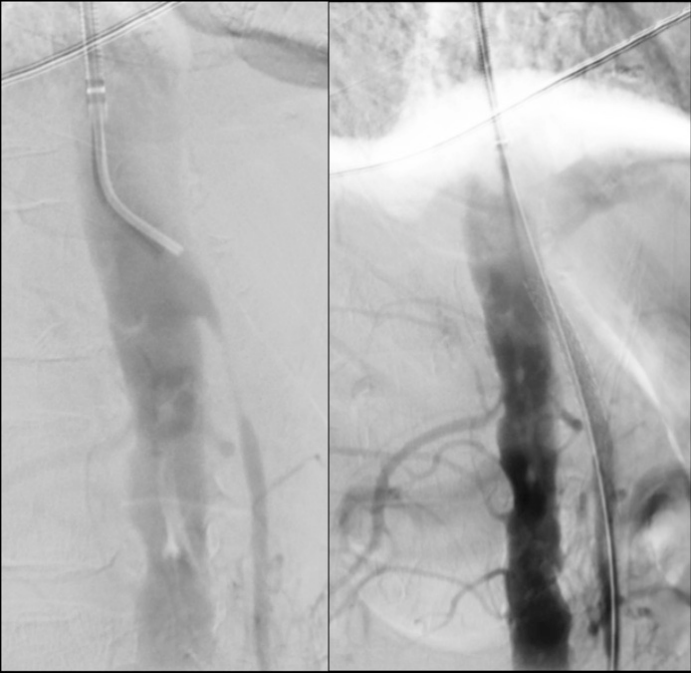
Angioplasty of the superior mesenteric artery with a covered balloon-expandable stent.

Biopsy ruled out neoplasm and *Helicobacter pylori*, with findings of mucosal erosion and coagulation and hemorrhagic necrosis, confirming ischemic gastritis. Clinical treatment was initiated with pantoprazole, simvastatin, and cilostazol. Endovascular treatment was scheduled to perform SMA angioplasty with a V12 6 x 38 mm stent (Figure 4) via a left brachial access. The patient was discharged on the 2nd postoperative day, on aspirin, with symptomatic improvement.

**Figure 5:**
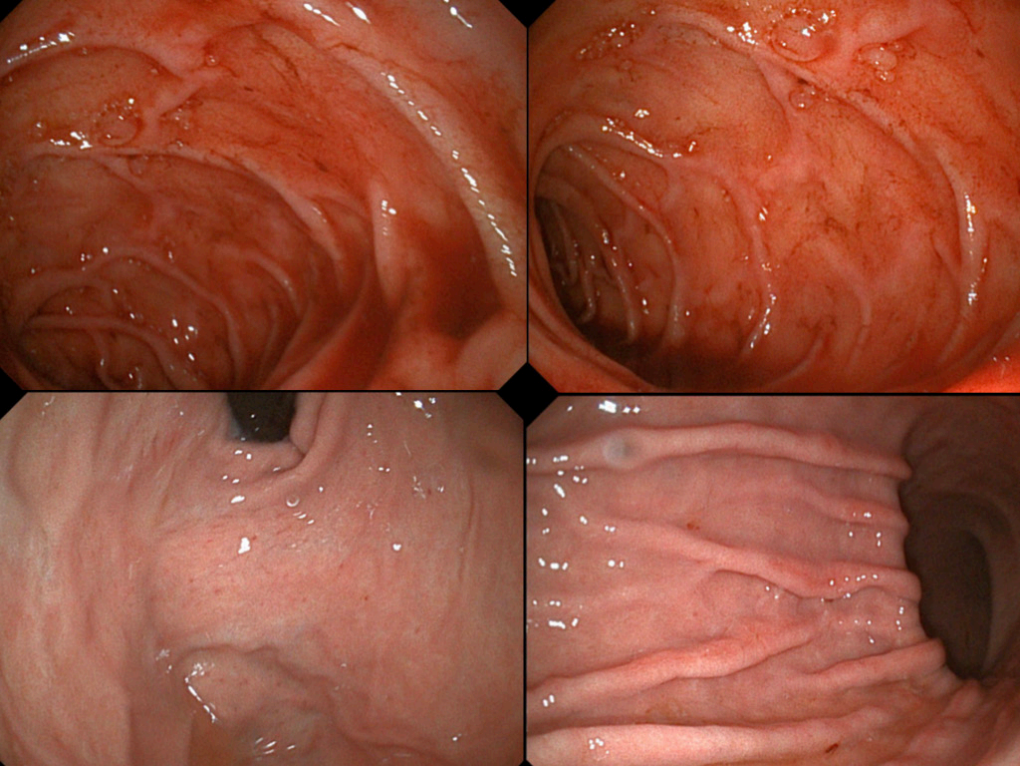
Control endoscopy showing healed ulcers and moderate pangastritis.

**Figure 6:**
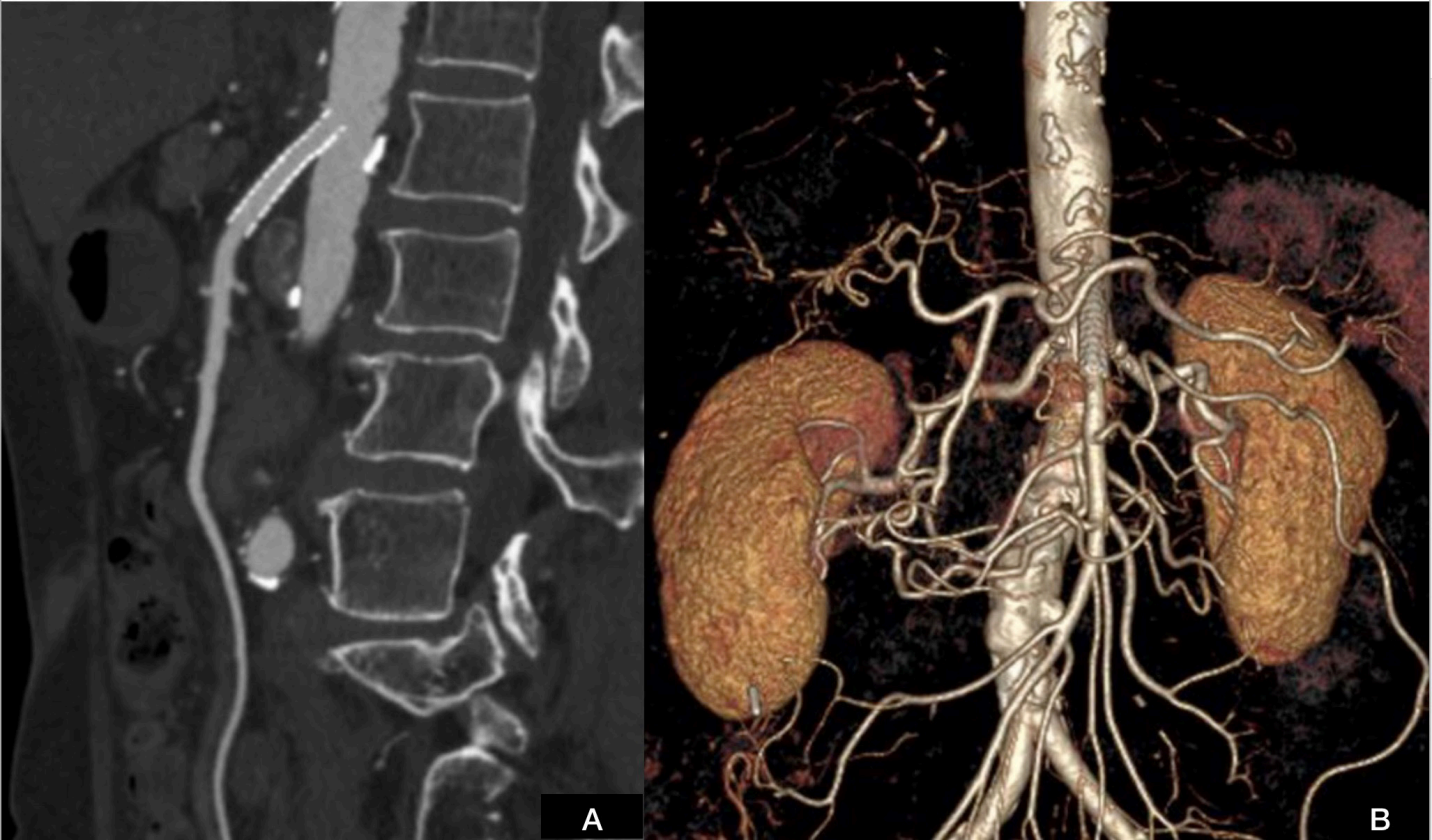
(A) Control angiotomography at 30 days, showing patent stent; and (B) control angiotomography at 6 months, with patent stent.

On the 8th postoperative day, the patient had already recovered 2 kg in weight and no longer had abdominal pains or bleeding, and clopidogrel was added to her treatment. At 30 days, UDE was performed, showing healed ulcers and moderate pangastritis (Figure 5), and control angiotomography showed a patent stent (Figure 6A). Six-months after treatment, the patient remains in good clinical condition and is asymptomatic. She has gained 10 kg in weight and the mesenteric stent remains patent and free from stenosis (Figure 6B).

## DISCUSSION

Gastric ischemia is a rare disease because the stomach has a vast collateral network, conferring considerable tolerance of central obstructions.^1-3^ The CTr is the principal supply of blood to the stomach, and the first branch of the SMA, the inferior pancreaticoduodenal artery, provides collateral circulation, as described by Rio Branco and Bühler.^1,2^ Collateral blood supply can also come via the esophageal and left inferior phrenic arteries.^1,2^ Rates of CTr or SMA occlusion discovered as incidental findings can be as high as 18%, but cases with clinical manifestations are uncommon.^2,3^

Also known as abdominal angina, chronic ischemia is one of the less common causes of mesenteric ischemia (5%) and is generally related to obstruction of two or three mesenteric vessels, although in 9% it may involve a single vessel.^4-6^ Gastric ischemia is very infrequent, and colonic ischemia is more common, affecting the angles of the splenic flexure and the rectosigmoid colon, which can be on the right in up to 40% of cases.^1,2^

The classic triad of symptoms of chronic mesenteric ischemia are postprandial abdominal pains, weight loss, and abdominal murmur.^5^ This is typically epigastric or mesogastric, with onset 20 to 30 minutes after eating and duration of 1 to 2 hours, and is associated with reduced food intake and weight loss, in addition to gastrointestinal, bleeding, which can occur in up to 47% of cases.^2^ In acute gastric ischemia, the entire stomach thickness is involved, with histopathological changes of coagulation necrosis, superficial erosions, necroinflammatory exudate, and hemorrhagic necrosis.^1^ In the chronic form, there are no distinctive histological changes and so biopsy is not of use to support diagnosis.^1^

Endoscopy helps in ruling out malignancy and is the most sensitive method for detecting early ischemic changes.^4,5^ Isolated findings of gastritis ischemic, duodenitis and, colitis are nonspecific, and ulcers in uncommon sites may be suggestive of ischemia gastric, after ruling out malignancy and use of anti-inflammatories and with a negative *Helicobacter pylori* test.^2,3^ Chronic gastric ischemia is infrequent and tends to be associated with bleeding ulcers along the large curvature of the posterior wall, which are very often multiple, irregularly shaped, with white sclerotic bases, and surrounded by erythematous and mottled mucosa.^4,7^

Doppler ultrasound is the first screening examination to use for diagnosis,^2,3^ with the following criteria: stenosis exceeding 70%: peak systolic velocity exceeding 275 cm/s in the SMA and exceeding 200 cm/s in the CTr.^2,3^ This finding has 92% sensitivity and 96% specificity for SMA stenosis.^2,3^ Occlusion of the CTr may also be suspected if there is reverse flow in the hepatic or splenic artery.^2,3^

Arteriography is considered the gold standard for diagnosis, with significant SMA stenosis defined by a mean blood pressure gradient greater than 10 mmHg.^2,3^ Angiotomography is the method of choice, supporting both topographic diagnosis and surgical planning and identifying cases of thrombosis, diffuse calcification, and tumors.^2,3,7^ Sensitivity and specificity are 96 and 94%, respectively.^2^ It is also superior to angiomagnetic resonance imaging because of its better spatial resolution and its capacity for assessment of occlusion of distal vessels, in addition to the shorter duration of the examination.^2^

For the acute form, initial management includes treatment to suppress gastric acid and gastric decompression.^1,4^ Wide spectrum antibiotics are recommended for cases with pneumatosis or gas in the portal system.^1,4,5^ Conservative treatment is associated with 24% mortality by direct causation in 6 months, although recurrence of hemorrhage is low.^8^ Cases complicated by perforation, sepsis, or persistent bleeding, must be managed surgically, including with gastrectomy.^9^

For chronic cases, revascularization is indicated in symptomatic cases.^2,3^ There is no evidence of benefit from conservative management with long term parenteral nutrition and without intervention, with 100% mortality at 5 years.^2,3^ Objectives include relief from symptoms, improved quality of life, weight gain, and reduced risk of infarction.^2,3^ In asymptomatic cases, prophylactic intervention is controversial and is rarely indicated.^2,3^

Endovascular treatment as first choice is safe over the short and medium term, with 6% mortality compared to 13% for open approaches.^9,10^ It offers lower morbidity and complication rates and shorter length of hospital stay, although rates of symptoms and reinterventions are higher.^2,3^ The SMA is the primary target, primarily for shorter lesions (less than 10 cm), ostial lesions, or occlusions with little calcification or thrombosis.^2,3,4,11^ Use of stenting for CTr lesions is linked with a higher risk of restenosis and is avoided in cases with extrinsic compression by the median arcuate ligament.^2,3^ Interventions involving the CTr may be considered if recanalization of the SMA fails.^2,3,11^ Simultaneous angioplasty of the CTr and the SMA remains controversial, reserved for cases of severe gastric ischemia with poor collateral circulation between them.^2,3^

Use of balloon-expandable covered stents is recommended for restenosis or short calcified ostial stenosis because of their additional radial strength and precise deployment.^3,12^ Results for restenosis rates, symptom recurrence, and primary patency are better and uncovered stents should be reserved for long stenoses, intraluminal dissections, and when preserving collaterals.^2,3,12^

Surgical revascularization is preferred for patients with low surgical risk and anatomy that is unfavorable for the endovascular method, for failed percutaneous treatment, and for intra-stent restenosis.^2,3^ Techniques include visceral artery endarterectomy and bypasses with veins or prosthetic grafts.

We highlight the importance of a multidisciplinary team, once duodenal neoplasm has been ruled out. It is suggested that endovascular treatment is the ideal method and is safe, especially when using covered stents in proximal and short mesenteric lesions. Treatment should be individualized, achieving high success rates, lower morbidity and mortality, and good patency over the medium term.
