# Supplementary material for: Severe ischemic gastritis caused by chronic mesenteric ischemia
Source: J Vasc Bras. 2023 Aug 11;22:e20230002. doi: 10.1590/1677-5449.202300022 (PMC10545228; doi:10.1590/1677-5449.202300022)
Supplement: Translation

# Translation: Gastrite isquêmica grave por isquemia mesentérica crônica

**DOI:** 10.1590/1677-5449.202300021

**Authors:** Bruno Moraes Ribas, Eduardo Camargo Rebolho, Guilherme Figueiró Ferronatto, Pedro Henrique Bragato, Hugo Genki Kagawa Akahane, Eduardo José Brommelstroet Ramos, Thienis Maria da Costa Lima, Barbara D’agnoluzzo Moreira

**Affiliations:** 1 Hospital Nossa Senhora das Graças - HNSG, Curitiba, PR, Brasil.; 2 Hospital Marcelino Champagnat, Curitiba, PR, Brasil.; 3 Hospital São Vicente - HSV, Curitiba, PR, Brasil.; 4 Universidade Federal do Paraná - UFPR, Curitiba, PR, Brasil.; 5 Hospital de Clínicas de São Paulo - HC-SP, São Paulo, SP, Brasil.

**Keywords:** isquemia mesentérica, oclusão vascular mesentérica, aterosclerose, procedimentos endovasculares, gastrite

## Abstract

A gastrite isquêmica é uma doença rara, podendo ocorrer por insuficiência vascular focal ou sistêmica. Essa condição é raramente vista na prática médica devido à vasta rede colateral arterial do estômago pelo tronco celíaco e mesentérica superior. A apresentação clássica da isquemia crônica é formada pela tríade de dor pós-prandial, perda de peso e sopro abdominal. A intervenção está indicada naqueles pacientes sintomáticos, sendo o tratamento endovascular uma alternativa à cirurgia em pacientes com alta comorbidade, tendo bons resultados. Reportamos um caso de gastrite isquêmica grave com úlceras e sangramento que foi causado por isquemia mesentérica crônica, em uma paciente de 71 anos, com oclusão do tronco celíaco e mesentérica inferior, além de estenose crítica da superior. O diagnóstico foi confirmado por exame de imagem, e a paciente foi submetida a tratamento endovascular. Trata-se de uma condição rara de diagnóstico e tratamento desafiadores, a qual requer uma equipe multidisciplinar para o manejo adequado.

## INTRODUÇÃO

A isquemia mesentérica pode ser secundária à insuficiência arterial, venosa ou não oclusiva^1^ . Os fatores predisponentes incluem doença cardiovascular, tabagismo, fibrilação arterial, hipercoagulabilidade e vasculites^2^ .

A forma aguda é definida como a interrupção abrupta do suprimento sanguíneo, sendo a embolia da artéria mesentérica superior (AMS) a mais comum (50%)^2^ . O sintoma é dor abdominal intensa, desproporcional ao exame físico, com início em minutos nos casos embólicos e em horas nas aterotrombóticas^2^ . Na progressão, as manifestações sistêmicas tornam-se proeminentes, com taquicardia, hipotensão, febre e leucocitose^2^ .

A forma crônica ocorre quando pelo menos duas das principais artérias estão comprometidas^2^ . É acometido principalmente o sexo feminino, de 40 a 70 anos, e há evidências de doença aterosclerótica em 2/3 dos casos^2^ . Ela é muitas vezes associada a perda de peso por “receio” de ingerir alimentos, podendo estar associada a náusea, vômitos, sangramento da mucosa e anemia^2^ .

A gastrite isquêmica é uma de suas raras apresentações, podendo ter etiologia de insuficiência vascular local ou sistêmica. A aterosclerose com acometimento do tronco celíaco é a causa mais comum (75%)^2,3^ . Das causas sistêmicas, 25% dos casos devem-se a hipoperfusão, com destaque para o choque séptico^2,3^ . O tratamento cirúrgico é o indicado nos casos sintomáticos, permitindo a mudança da evolução natural e um melhor prognóstico^1^ .

O protocolo foi aprovado pelo comitê de ética de nossa instituição (CAAE:60299722.8.0000.0096, parecer nº 5.697.538).

## DESCRIÇÃO DO CASO

Paciente feminina, 71 anos, 37 kg, com queixa de dor abdominal tipo cólica, diarreia crônica e emagrecimento de aproximadamente 10 kg em 1ano. A dor ocorria 2-3 vezes ao dia, sendo mais intensa em hipocôndrio direito, e não possuía relação estrita com a alimentação. A paciente era hipertensa e ex-tabagista, tendo cessado há 20 anos, com histórico de colecistectomia aberta e apendicectomia. Ela apresentava-se emagrecida, com abdome escavado, flácido e indolor, sem massas palpáveis e com presença de sopro sistólico em epigástrio.

Na tomografia de abdome com contraste, foi evidenciado nódulo hipodenso em ampola duodenal de 5 mm (Figura 1). Houve oclusão do tronco celíaco (TrC), porém com reenchimento distal por colaterais, oclusão da mesentérica inferior e estenose crítica em AMS, com 1 mm de luz (Figura 2). Diante da hipótese de carcinoma da ampola duodenal ou de vias biliares, ela foi submetida a endoscopia digestiva alta (EDA), que demonstrou múltiplas úlceras gástricas superficiais de bordas irregulares, sangrantes ao longo do corpo do estômago, com áreas de palidez, sugestivas de gastrite isquêmica e papila duodenal normal (Figura 3).

**Figura 1:** Tomografia de abdome com nódulo hipotensão de 5 mm em ampola duodenal.

**Figura 2:** Angiotomografia de aorta abdominal e ramos, com oclusão do tronco celíaco com preenchimento distal por colaterais, estenose crítica em artéria mesentérica superior e artéria mesentérica inferior.

**Figura 3:** Endoscopia digestiva alta evidenciando múltiplas úlceras sangrantes superficiais, com bordas irregulares e áreas de palidez.

Com a biópsia, foram descartadas neoplasia e *Helicobacter pylori*, com achados de erosão da mucosa, necrose coagulativa e hemorrágica, confirmando a gastrite isquêmica. Foi iniciado tratamento clínico com pantoprazol, sinvastatina e cilostazol. Programou-se o tratamento endovascular com angioplastia da AMS com implante de *stent* V12 6 x 38 mm (Figura 4) por acesso via artéria braquial esquerda. A paciente recebeu alta no 2° dia, com ácido acetilsalicílico e melhora dos sintomas.

**Figura 4:** Angioplastia da artéria mesentérica superior com *stent* recoberto balão-expansível.

No 8° dia pós-operatório, a paciente já havia recuperado 2 kg e não apresentava mais dor abdominal nem sangramentos, optando-se por acrescentar clopidogrel. Em 30 dias, foram realizadas EDA e angiotomografia de controle, com úlceras cicatrizadas e pangastrite moderada (Figura 5) e perviedade do *stent* (Figura 6A). Após 6 meses do tratamento, a paciente permanece bem clinicamente, assintomática, com recuperação de 10 kg e com *stent* mesentérico pérvio e sem estenoses (Figura 6B).

**Figura 5:** Endoscopia de controle com úlceras cicatrizadas e pangastrite moderada.

**Figura 6:** (A) Angiotomografia de controle após 30 dias, com *stent* pérvio; e (B) angiotomografia de controle após 6 meses, com *stent* pérvio.

## DISCUSSÃO

A isquemia gástrica é uma doença rara, uma vez que o estômago conta com vasta rede colateral, permitindo uma tolerância significativa a obstruções centrais^1-3^ . O TrC provê o suprimento principal ao estômago, e o primeiro ramo da AMS, a artéria pancreatoduodenal inferior, fornece circulação colateral, como descrito por Rio Branco e Bühler^1,2^ . O suprimento colateral também pode vir das artérias esofágicas e frênica inferior esquerda^1,2^ . Os achados incidentais de oclusão de TrC ou AMS podem chegar a 18%, porém aquelas com manifestações clínicas são pouco comuns^2,3^ .

Também chamada de angina abdominal, a isquemia crônica é uma causa menos comum de isquemia mesentérica (5%), geralmente relacionada à obstrução de dois ou três dos vasos mesentéricos e, em até 9%, pode acometer um único vaso^4-6^ . É muito pouco frequente a ocorrência de isquemia gástrica, sendo mais comum a isquemia colônica, nas áreas das vertentes da flexura esplênica e do cólon retossigmoide, podendo estar à direita em 40% dos casos^1,2^ .

Os sintomas da tríade clássica da isquemia mesentérica crônica são dor abdominal pós-prandial, perda de peso e sopro abdominal^5^ . Ela é tipicamente epigástrica ou mesogástrica, com início após 20 a 30 minutos da alimentação e duração de 1 a 2 horas, e está associada à redução de ingesta alimentar e perda de peso, além de sangramento gastrointestinal, que pode chegar a 47% dos casos^2^ . Na isquemia gástrica aguda, há envolvimento da espessura total do estômago, com alterações histopatológicas de necrose coagulativa, erosões superficiais, exsudato necroinflamatório e necrose hemorrágica^1^ . Já na forma crônica, não há alterações histológicas discriminativas, de modo que a biópsia não tem valor para apoiar o diagnóstico^1^ .

A endoscopia auxilia na exclusão de malignidade e é o método mais sensível para detectar alterações isquêmicas precoces^4,5^ . Os achados isolados de gastrite isquêmica, duodenite e colite são inespecíficos, podendo ser sugestivas de isquemia gástrica as úlceras em locais incomuns, excluindo malignidade, uso de anti-inflamatórios e teste de *Helicobacter pylori* negativo^2,3^ . A isquemia gástrica crônica é pouco frequente e costuma estar associada a úlceras sangrantes ao longo da grande curvatura da parede posterior, sendo, muitas vezes, múltiplas e de formato irregular, com base esclerótica esbranquiçada, circundadas por mucosa eritematosa e mosqueada^4,7^ .

O Doppler é o primeiro exame de rastreamento para o diagnóstico^2,3^ , com os critérios de estenose sendo maiores que 70%: velocidade de pico sistólico maior que 275 cm/s na AMS e maior que 200 cm/s no TrC^2,3^ . Apresenta-se uma sensibilidade de 92%, além de especificidade de 96% para estenose da AMS^2,3^ . A oclusão do TrC pode ainda ser sugerida quando há uma inversão do fluxo nas artérias hepáticas ou esplênicas^2,3^ .

A arteriografia é considerada o padrão-ouro de diagnóstico, com a estenose significativa da AMS definida por um gradiente de pressão arterial média maior que 10 mmHg^2,3^ . A angiotomografia é o método de escolha, auxiliando no diagnóstico topográfico e no planejamento cirúrgico, identificando casos de trombose, calcificação difusa e tumor^2,3,7^ . A sensibilidade e especificidade são de 96 e 94%, respectivamente^2^ . Além disso, ela é superior à angioressonância devido à melhor resolução espacial e avaliação de oclusões de vasos distais, além do menor tempo de exame^2^ .

Na forma aguda, o tratamento inicial inclui a terapia supressora de ácido gástrico e descompressão gástrica^1,4^ . O uso de antibiótico de amplo espectro é recomendado naqueles casos com pneumatose ou gás no sistema porta^1,4,5^ . O tratamento conservador apresenta mortalidade por causa direta de 24% em 6 meses, embora a recorrência de hemorragia seja baixa^8^ . Os casos complicados, com perfuração, sepse ou sangramento persistente, devem ser manejados cirurgicamente, incluindo gastrectomia^9^ .

Nos casos crônicos, a revascularização está indicada nos casos sintomáticos^2,3^ . Não há evidências de benefícios da abordagem conservadora com nutrição parenteral de longo prazo e não intervencionista, com mortalidade de 100% em 5 anos^2,3^ . O objetivo inclui o alívio dos sintomas, a melhora da qualidade de vida, ganho de peso e diminuição do risco de infarto^2,3^ . Nos casos assintomáticos, a intervenção profilática é controversa e raramente indicada^2,3^ .

O tratamento endovascular como primeira escolha é seguro a curto e médio prazo, com mortalidade de 6%, em contrapartida à aberta, de 13%^9,10^ . Ele oferece menores morbidade, taxa de complicações e tempo de internação, apesar da maior recorrência dos sintomas e reintervenções^2,3^ . A AMS é o maior alvo, principalmente nas lesões curtas (menos de 10 cm), ostiais ou oclusões com pouca calcificação ou trombose^2,3,4,11^ . Nas lesões do TrC, o uso de *stent* apresenta maior risco de reestenose, sendo evitado nas compressões extrínsecas pelo ligamento arqueado mediano^2,3^ . As intervenções do TrC podem ser consideradas na falha na recanalização da AMS^2,3,11^ . A angioplastia simultânea do TrC e da AMS permanece controversa, reservada nos casos de isquemia gástrica severa e pobre circulação colateral entre as mesmas^2,3^ .

Recomenda-se o uso de *stents* balão-expansíveis revestidos para reestenoses ou estenoses curtas calcificadas ostiais devido à maior força radial e liberação precisa^3,12^ . Há melhores resultados na taxa de reestenose, recorrência dos sintomas e patência primária, reservando os *stents* não revestidos para estenoses longas, dissecções intraluminais e para preservar as colaterais^2,3,12^ .

A revascularização cirúrgica é preferencial nos pacientes com baixo risco cirúrgico e anatomia desfavorável para o método endovascular, com falha no tratamento percutâneo e reestenoses intra-*stent* ^2,3^ . As técnicas incluem a endarterectomia da artéria visceral, derivação com enxerto de veia ou protética.

Destacamos a importância de se ter uma equipe multidisciplinar, uma vez descartada a neoplasia duodenal. Sugere-se que o tratamento endovascular seja um método ideal e seguro, especialmente com o uso de *stents* revestidos nas lesões mesentéricas proximais e curtas, devendo ser trado de forma individualizada, com altas taxas de sucesso, menor morbimortalidade e boa perviedade a médio prazo.
